# Multimorbidity, Loneliness, and Social Isolation. A Systematic Review

**DOI:** 10.3390/ijerph17228688

**Published:** 2020-11-23

**Authors:** André Hajek, Benedikt Kretzler, Hans-Helmut König

**Affiliations:** Department of Health Economics and Health Services Research, University Medical Center Hamburg-Eppendorf, Martinistraße 52, 20246 Hamburg, Germany; b.kretzler.ext@uke.de (B.K.); h.koenig@uke.de (H.-H.K.)

**Keywords:** multimorbidity, multiple chronic conditions, loneliness, social frailty, social isolation, social exclusion, loneliness, chronic diseases, COVID-19, SARS-CoV-2

## Abstract

No systematic review has appeared so far synthesizing the evidence regarding multimorbidity and loneliness, social isolation, or social frailty. Consequently, our aim was to fill this gap. Three electronic databases (PubMed, PsycINFO, and CINAHL) were searched in our study. Observational studies examining the link between multimorbidity and loneliness, social isolation, and social frailty were included, whereas disease-specific samples were excluded. Data extraction included methods, characteristics of the sample, and the main results. A quality assessment was conducted. Two reviewers performed the study selection, data extraction, and quality assessment. In sum, eight studies were included in the final synthesis. Some cross-sectional and longitudinal studies point to an association between multimorbidity and increased levels of loneliness. However, the associations between multimorbidity and social isolation as well as social frailty remain largely underexplored. The quality of the studies included was rather high. In conclusion, most of the included studies showed a link between multimorbidity and increased loneliness. However, there is a lack of studies examining the association between multimorbidity and social isolation as well as social frailty. Future studies are required to shed light on these important associations. This is particularly important in times of the COVID-19 pandemic.

## 1. Introduction

There are several similar concepts concentrating on the social needs of individuals [1]. Specifically, social isolation, loneliness, and social frailty exist. While social isolation can be defined as the feeling that an individual does not belong to the society [2], loneliness refers to the feeling that an individual’s social network is smaller or of poorer quality than preferred [2], and social frailty refers to the lack of resources to fulfill one’s basic social needs [1]. Given the fact that traditional family bonds become ruptured, new challenges arise for individuals. It should be emphasized that these social needs are associated with physical frailty and subsequent mortality [3,4]. In sum, these social needs have been considered as new geriatric giants [5]. Moreover, social needs can also have deleterious consequences for younger individuals.

Previous studies have determined several factors associated with these social needs. For example, it has been shown that they are, among other things, associated with income poverty or experiencing a fall [6,7]. Moreover, it has been shown that they are associated with multimorbidity (e.g., [8,9,10]),

Multimorbidity is commonly defined as the existence of at least two chronic illnesses [11]. The prevalence of multimorbidity is rather high in adults, especially in very old individuals [12,13]. According to a systematic review, the prevalence of multimorbidity in older individuals ranges from 55% to 98% [14]. The prevalence also increases in women and people from low social classes [14]. Little is known about the genetic and biological risk factors for multimorbidity [14]. In light of the demographic aging in high-income countries, it is projected that the number of individuals with multimorbidity will increase. Multimorbidity is also linked to disability [14], mortality [15], and high health care costs [16].

While some observational studies exist examining the link between multimorbidity and social needs (in terms of social isolation, loneliness, and social frailty) [8,9,10], there is a lack of a study systematically synthesizing observational studies investigating these associations. Thus, our objective of this systematic review was to fill this gap in knowledge.

Particularly in times of the COVID-19 pandemic, knowledge about the link between multimorbidity and loneliness, social isolation, or social frailty is of great importance. This can be explained by the fact that the case fatality rate increases considerably with age. Consequently, older adults are quite often forced to avoid physical contact to stay at home.

## 2. Materials and Methods

This systematic review was conducted in accordance with the Preferred Reporting Items for Systematic Reviews and Meta-Analysis Protocols guidelines [17] and is registered with the International Prospective Register of Systematic Reviews (PROSPERO, registration number: CRD42020179918).

### 2.1. Search Strategy and Selection Criteria

In July and August 2020, a systematic literature search was conducted based on three databases (Medline, PsycINFO, and CINAHL). In Table 1, the search query for Medline is depicted.

Two reviewers (AH, BK) evaluated the studies for inclusion/exclusion using a two-step process. First, a title/abstract screening was performed. Second, a full-text screening was conducted. Furthermore, we hand searched the reference lists of studies selected for inclusion. If disagreements occurred, we used discussions to resolve it (and, if required, included a third party (HHK)).

We had the following inclusion criteria:

- Cross-sectional and longitudinal observational studies investigating the association between (1) multimorbidity and social frailty, or (2) multimorbidity and loneliness, or (3) multimorbidity and social isolation.
- Studies appropriately quantifying important variables like social isolation.
- Studies published in peer-reviewed journals (English or German language).

Exclusion criteria were:

- Studies not investigating the association between (1) multimorbidity and social frailty, or (2) multimorbidity and loneliness, or (3) multimorbidity and social isolation.
- Studies exclusively investigating samples with a specific disorder.
- Study design other than observational.
- Inappropriate assessment of important variables.

Selection criteria did not include any restrictions regarding place and time during which studies were conducted. Using a sample of 100 titles/abstracts, we conducted a pre-testing of eligibility criteria. Results of this pre-testing did not affect the final eligibility criteria list.

### 2.2. Data Extraction and Analysis

One reviewer (BK) conducted the data extraction. A second reviewer (AH) cross-checked the extracted data. If disagreements occurred, discussions were held to reach a consensus. If required, a third party (HHK) was included. If clarification was needed, we contacted the study authors.

Data extraction covered study design, measures, analytical approach, description of the sample, and key results. We present the key results as follows (in each case: (i) cross-sectional, and (ii) longitudinal):

(1) multimorbidity and loneliness.
(2) multimorbidity and social isolation.
(3) multimorbidity and social frailty.

### 2.3. Quality Assessment

The study quality was assessed independently by two reviewers (AH, BK) based on the well-known and widely used NIH Quality Assessment Tool for Observational Cohort and Cross-Sectional Studies [18]. In case of disagreement, discussions were held to resolve the conflict. A third party (HHK) was included in such discussions as needed.

## 3. Results

This section is divided by subheadings. It provides a concise and precise description of the experimental results, their interpretation as well as the experimental conclusions that can be drawn.

### 3.1. Overview of Included Studies

**Figure 1:**
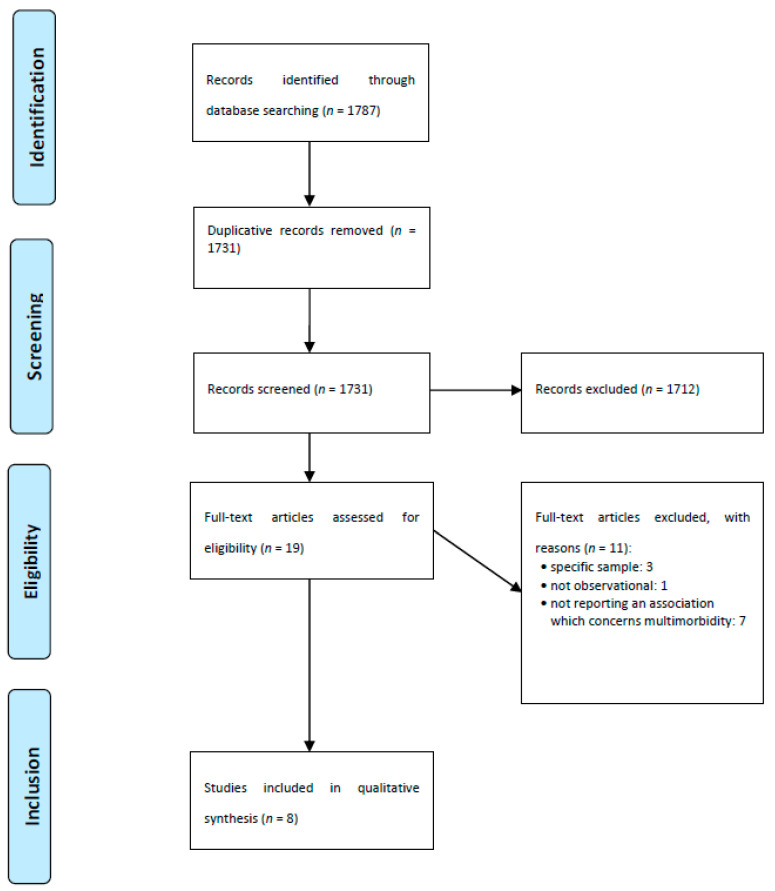
Flow chart.

The study selection process is shown in Figure 1 [19]. In sum, *n* = 8 studies were included in the final synthesis of our review. Important characteristics and key results of the studies included are given in Table 2. If reported, adjusted results are displayed.

Data stemmed from Europe (*n* = 6, with two studies from Germany, and one study each from Denmark, Netherlands, Spain and the United Kingdom) and North America (*n* = 2 studies from Canada). Equally, four cross-sectional and four longitudinal studies were identified. The observation period in the longitudinal studies varied from three to twelve years. It should be noted that while one study used cross-sectional data from the German Aging Survey (year 2014) [20], the second longitudinal study used data from 2002 to 2014 from the German Aging Survey [9]. Multimorbidity was commonly defined as having two or more chronic conditions.

One study reported on data from individuals recruited from a general practice [21], another analyzed data from a heterogeneous sample of community-dwelling older adults [22], and all others conveyed results from large, nationally representative samples of community-dwelling older adults. The sample size ranged from 121 to 36,397 individuals, the proportion of women in the samples ranged from 49% to 56%, and the average age ranged from 60 to 77 years. Further details are given in Table 2.

In the next sections, key results are presented as follows (in each case: (i) cross-sectional, and (ii) longitudinal):

(1) multimorbidity and loneliness.
(2) multimorbidity and social isolation.
(3) multimorbidity and social frailty.

### 3.2. Multimorbidity and Loneliness

With regard to cross-sectional studies, five studies examined the link between multimorbidity and loneliness [20,21,23,24,25]. Three out of these five studies found a positive association between multimorbidity and loneliness. In contrast, one study did not find a bivariate association between multimorbidity and loneliness [24], and another study did not identify such a link using multiple regressions [21].

With regard to sex differences, one cross-sectional study [25] showed that loneliness was associated with multimorbidity in middle-aged and older (i.e., 45 to 54 years, 55 to 64 years, 65 to 74 years and 75+) men and women in Canada and Australia (except for Australian men aged 75+). However, this study did not include interaction terms to test whether potential sex differences were significant [25]. The remaining studies [20,21,23,24] only adjusted for sex.

With regard to longitudinal studies, three studies examined this link [9,22,26]. All of these studies found a link between multimorbidity and increased loneliness scores longitudinally. Sex differences were not examined.

### 3.3. Multimorbidity and Social Isolation

With regard to cross-sectional studies, only one study examined the link between multimorbidity and social isolation [9]. This study found an association between multimorbidity and increased social isolation. In contrast, there was a lack of longitudinal studies investigating the link between multimorbidity and social isolation. Sex differences were not examined.

### 3.4. Multimorbidity and Social Frailty

Our systematic review did not identify either cross-sectional or longitudinal studies examining the link between multimorbidity and social frailty.

### 3.5. Quality Assessment

The evaluation of study quality of the included studies is shown in Table 3. While some criteria were fulfilled by all studies (e.g., adjustment for important covariates), some other criteria were only fulfilled by a few studies (e.g., response rate ≥50%). However, the general study quality was rather high. More precisely, the study quality of five studies were rated as ‘good’ and three studies were rated as ‘fair’, which also means that none of the studies were rated as ‘poor’.

## 4. Discussion

In sum, eight studies were included in the final synthesis. Some cross-sectional and longitudinal studies pointed to an association between multimorbidity and increased levels of loneliness. However, the associations between multimorbidity and social isolation as well as social frailty remain largely underexplored. The quality of the studies included was rather high. For example, several studies used data from nationally representative samples like the English Longitudinal Study of Aging [26] or the German Aging Survey [9].

The link between multimorbidity and loneliness appears to be plausible. For example, as stated by Barlow et al. [22], multimorbidity is associated with lower physical functioning, which may affect loneliness. However, this factor was commonly adjusted for in the studies examined. Another possible explanation may be that loneliness is rather associated with the quality of the relationships, but not with the quantity [27]. This means that multimorbidity may affect loneliness by reducing the relationship quality [9]. In the same vein, a qualitative study demonstrated that the social networks among individuals with multimorbidity were rather large and diverse (including health care professionals) [28]. These presumably one-sided relationships to health care professionals may reflect a decreased relationship quality among individuals with multimorbidity [9]. Jessen et al. [23] provided an additional explanation: Individuals with multimorbidity have to cope with symptoms and have frequent contact with the health care system, which can restrict participation in social activities [29]. Moreover, individuals with multimorbidity may leave the labor market, which can markedly reduce the everyday contact with colleagues [23].

Olaya et al. [24] provided two possible explanations for the association between social needs and multimorbidity. First, according to the buffering hypothesis, social needs can buffer the negative impact of stress on health [30]. Moreover, another explanation may be that social factors can assist in regulating health behavior and can increase the access to health care (e.g., transportation or financial support) [31]. Equal explanations are given by Singer et al. [26] and Jessen et al. [23]. Additionally, Jessen et al. stated that loneliness can cause emotional changes, which in turn affect multimorbidity [32]. These emotional changes can activate neurobiological and behavioral mechanisms that can decrease health [32].

Depending on the proposed directionality, conclusions in the included studies varied from (i) proposing efforts to decrease loneliness to reduce multimorbidity [26] to (ii) tackling multimorbidity to reduce loneliness [9]. Moreover, (iii) the need for future, longitudinal studies [20] and (iv) studies elucidating the underlying mechanisms was stressed [9].

The comparability of the included studies was somewhat restricted. Different tools were used to assess loneliness scores. For instance, while some studies used the De Jong Gierveld scale [33], other studies used the UCLA scale [34]. Both scales conceptualize loneliness as subjective. Nevertheless, while the UCLA scale views loneliness mainly as affective, the De Jong Gierveld scale views it as cognitive [35]. A previous study concluded that the latter scale might be a better choice for cross-sectional and longitudinal studies when focusing on middle-aged and older adults [35].

Both German studies used the 6-item version of the De Jong Gierveld loneliness scale [9,20]. These studies showed that multimorbidity was associated with increased loneliness both cross-sectionally and longitudinally. Apart from these studies, different tools (or different versions of the UCLA loneliness scale) were used to quantify loneliness.

Moreover, with regard to comparability, while multimorbidity was very consistently defined as the presence of two or more chronic conditions, the list of diseases ranged from eight to 25 diseases, which may have an impact on the results. Moreover, the assessment of chronic conditions mostly refers to self-ratings in the studies examined. The samples included were quite comparable with regard to the proportion of female individuals and age bracket (mainly including individuals in middle- and old age). Furthermore, there were some differences in the analytical approach used (for example, fixed effects regressions vs. the use of generalized estimating equations (GEE)), which in turn can have quite a large impact on the results [36]. For example, using fixed effects strategies when panel data are present may assist in identifying the link between the onset of multimorbidity and loneliness, social isolation, and social frailty [36].

Our systematic review identified possible gaps in knowledge. More precisely, there is a general gap in knowledge regarding the associations between (i) multimorbidity and social isolation (including tools to quantify “objective social isolation” [37]) and (ii) multimorbidity and social frailty. Moreover, as, for example, proposed by Kristensen et al. [20], the directionality between these factors should be further explored. It appears plausible that the onset of multimorbidity may increase feelings of e.g., social isolation. However, it also appears plausible that feelings of social isolation reduce, among other things, physical activities, which can in turn contribute to the occurrence of chronic illnesses or multimorbidity [9]. Future longitudinal studies using advanced methods like dynamic panel data estimation strategies [38] may assist in clarifying this issue. This knowledge may have important policy implications and, for example, may assist in reducing the social and economic burden caused by loneliness, social isolation, and social frailty. Furthermore, most studies used data from European countries. Therefore, future research is needed from other regions (like Asian, South American, or African countries). It may be the case that the link between multimorbidity and social needs is moderated by cultural background. Wister et al. [25] also proposed that future research should focus on different age group cohorts. Moreover, they proposed that the role of sex should be clarified [25], since men tended to be more stoic [39]. Furthermore, factors such as health literacy [40] (including social support for health [41]) or coping strategies such as flexible goal adjustment [42,43] may act as a moderator of the relationship between multimorbidity and social needs.

Additionally, the link between multimorbidity patterns or clusters (i.e., combination of (i) mental health problems, (ii) musculoskeletal disorders as well as (iii) cardiovascular and metabolic diseases) and social needs should be further explored in future studies [44].

Some strengths of our systematic review are worth highlighting. This is the first systematic review focusing on the link between multimorbidity and loneliness, social isolation, and social frailty. Key steps were performed by two reviewers (e.g., steps like study selection or data extraction). Furthermore, we conducted a quality assessment. Due to study heterogeneity, a meta-analysis was not performed. Due to the restriction to peer-reviewed articles, which ascertains a rather high quality, at least some previous findings (e.g., from grey literature) might be lacking. Moreover, due to the restriction to studies published in English or German language, relevant studies published in other languages (e.g., French language) were not included in this work.

## 5. Conclusions

Most of the included studies showed a link between multimorbidity and increased loneliness. However, there is a lack of studies examining the association between multimorbidity and social isolation as well as social frailty. Future studies are required to shed light on these important associations. This is particularly important in times of the COVID-19 pandemic. Upcoming studies should explore the role of factors such as social distancing or perceptions of safe practices in the link between multimorbidity and social needs.

## Figures and Tables

**Table 1:** Search strategy (PubMed).

#	Search Term
#1	Loneliness
#2	Social exclusion
#3	Social frailty
#4	Social isolation
#5	#1 OR #2 OR #3 OR #4
#6	Multimorbidity
#7	multiple chronic
#8	disease *
#9	condition *
#10	illness *
#11	#7 AND (#8 OR #9 OR #10)
#12	#6 OR #11
#13	#5 AND #12

Notes: Please note that the asterisk (“*”: in “disease*” (#8)) in PubMed is a truncation symbol. It can be used at the end of a word to search for all terms that begin with that basic root.

**Table 2:** Extracted data.

Study	Study Type/Time Span	Sample Source/Size	Age	Loneliness Assessment	Multimorbidity Assessment	Main Results	Quality Assessment Score
Barlow, M et al. (2014)	Longitudinal Five waves, from 2004 to 2012)	Montreal Aging and Health Study (Canada) *N* = 121 (56.2% females)	M = 71.2 SD = 4.7 64–83	Two items	Number of chronic illnesses (from a list of 17 diseases)	Growth-curve models showed that chronic illness was positively associated with loneliness (yearly change: ß = 0.125, *p* < 0.05).	Fair
Jessen, M et al. (2018)	Cross-sectional	National Longitudinal Survey of Ageing (Denmark) *N* = 9154 (54.3% females)	Not reported	UCLA Loneliness scale (20 items)	Two or more chronic conditions (from a list of eight diseases)	Logistic regression revealed that loneliness was positively associated with multimorbidity (OR = 1.77, 95% CI: 1.20–3.35).	Good
Kristensen, K. et al. (2019a)	Longitudinal Four waves, from 2002 to 2014	German Aging Survey (Germany) *N* = 12,692 (48.9% females)	M = 63.5 SD = 11.4	De Jong Gierveld short scales for loneliness (six items)	Two or more illnesses (from a list of 13 diseases)	Fixed effects regression stated that multimorbidity was associated with increased levels of loneliness (ß = 0.06, *p* < 0.001).	Good
Kristensen, K. et al. (2019b)	Cross-sectional	German Aging Survey (Germany) *N* = 7604 (53.6% females)	M = 59.8 SD = 10.6	De Jong Gierveld short scales for loneliness (six items)	Two or more illnesses (from a list of 13 diseases)	Linear regression detected a positive association between multimorbidity and loneliness (ß = 0.08, *p* < 0.001).	Good
Olaya, B. et al. (2017)	Longitudinal Two waves, from 2011/12 to 2014/15	Edad con Salud (Spain) *N* = 2113 (55.2% females)	M = 71.8 95% CI: 71.4–72.1	UCLA Loneliness scale (three items)	Number of chronic conditions (from a list of eight diseases)	Cox Proportional Hazard models did not find an association between multimorbidity on the one side and high loneliness (ref.: low loneliness) (ß = 0.003, *p* = 0.991) or high social support (ref.: low social support) (ß = 0.69, *p* = 0.262) on the other side.	Good
Renne, I & Gobbens, R. (2018)		Recruited from a general practice (The Netherlands) *N* = 241 (48.9% females)	M = 76.5 SD = 5.1 70–90	Assessment of social domain of frailty (TFI (three items))	Number of chronic conditions (from a list of nine diseases)	Linear regression showed that multimorbidity was negatively associated with quality of life (ß = -3.786, *p* < 0.001).	Fair
Singer, L. et al. (2019)	Longitudinal Seven waves from 2002 to 2014	English Longitudinal Study of Ageing (United Kingdom) *N* = 15,046 (55.3% females)	M = 66.0 SD = 10.9	One item	Basic multimorbidity: two or more morbidities (from a list of 25 diseases) Complex multimorbidity: three or more body systems affected	Generalized Estimating Equations revealed that multimorbidity was positively associated with low household wealth (ref.: high) (OR = 1.47, 95% CI: 1.34–1.61), a low subjective social status (ref.: high) (OR = 1.14, 95% CI: 1.04–1.24), a semi/routine occupation (ref.: manager, professional) (OR = 1.07, 95% CI: 1.04–1.24), a low sense of control (ref.: high) (OR = 1.57, 95% CI: 1.41–1.74), having no friends (ref.: very/some supportive friends) (OR = 1.14, 95% CI: 1.02–1.26), having no partner (ref. very/some supportive partner) (OR = 1.15, 95% CI: 1.06–1.26) and loneliness (OR = 1.19, 95% CI: 1.11–1.28).	Fair
Wister, A. et al. (2016)	Cross-sectional	Canadian Community Health Survey (Canada) and Household, Income and Labor Dynamics in Australia (Australia) *N* = 36,397 (51.9% females)	45–54: 38.1% 55–64: 29.7% 65–74: 17.9% ≥75: 14.3%	Hughes et al. 3-item loneliness scale	Number of chronic illnesses (from a list of eight diseases)	OLS regression showed that there was a significant positive association between multimorbidity and loneliness for all combinations of age group, gender and country, except Australian men which were older than 75 (ß = 0.02, 95% CI: −0.14–0.17).	Good

Notes: M = mean; SD = standard deviation; OR = odds ratio; OLS = ordinary least squares; TFI = Tilburg Frailty Indicator; UCLA = University of California, Los Angeles; Barlow et al. (2014): adjusted for age, female, socio-economic status and partnership status, and health engagement strategies as well as health-related self-protection; Jessen et al. (2018): adjusted for sex, year of birth, marital status, cohabitation status, attachment to the labor market, and home ownership; Kristensen et al. (2019a): adjusted for age, BMI, depressive symptoms, monthly net equivalent income, physical activity, self-rated health, marital status, and employment status; Kristensen et al. (2019b): adjusted for sex, age, marital status, monthly net equivalent income, BMI, depressive symptoms, current smoking status, alcohol consumption and physical activity; Olaya et al. (2017): adjusted for social support, loneliness, smoking, age, years of education, marital status, alcohol consumption, and depression; Renne & Gobbens (2018): adjusted for sex, age, marital status, education, and 15 frailty components from the Tilburg Frailty Indicator; Singer et al. (2019): adjusted for participation, sense of control, supportive children, supportive friends, and supportive partner; Wister et al. (2016): adjusted for marital status, foreign-born status, and education level.

**Table 3:** Quality assessment.

Questions	Studies							
	Barlow (2014)	Jessen (2018)	Kristensen (2019a)	Kristensen (2019b)	Olaya (2017)	Renne (2018)	Singer (2019)	Wister (2016)
1. Was the research question or objective in this paper clearly stated?	yes	yes	yes	yes	yes	yes	yes	yes
2. Was the study population clearly specified and defined?	yes	yes	yes	yes	yes	yes	yes	yes
3. Was the participation rate of eligible persons at least 50%?	not reported	yes (73.5%)	no (27.1%–50.3%)	no (27.1%)	yes (69.9%)	no (47.5%)	not reported	not reported
4. Were all the subjects selected or recruited from the same or similar populations (including the same time period)? Were inclusion and exclusion criteria for being in the study prespecified and applied uniformly to all participants?	yes	yes	yes	yes	yes	yes	yes	yes
5. Was a sample size justification, power description, or variance and effect estimates provided?	no	no	no	no	no	no	no	no
6. For the analyses in this paper, were the exposure(s) of interest measured prior to the outcome(s) being measured? (if not prospective should be answered as ‘no’, even is exposure predated outcome)	yes	no (cross-sectional)	no (simultaneously)	no (cross-sectional)	no (simultaneously)	no (cross-sectional)	no (simultaneously)	no (cross-sectional)
7. Was the timeframe sufficient so that one could reasonably expect to see an association between exposure and outcome if it existed?	yes	no (cross-sectional)	yes	no (cross-sectional)	no	no (cross-sectional)	yes	no (cross-sectional)
8. For exposures that can vary in amount or level, did the study examine different levels of the exposure as related to the outcome (e.g., categories of exposure, or exposure measured as continuous variable)?	dichotomous and continuous	dichotomous	dichotomous	dichotomous	dichotomous	continuous	dichotomous	continuous
9. Were the exposure measures (independent variables) clearly defined, valid, reliable, and implemented consistently across all study participants?	yes	yes	yes	yes	yes	yes	yes	yes
10. Was the exposure(s) assessed more than once over time?	no	no	yes	no	no	no	yes	no
11. Were the outcome measures (dependent variables) clearly defined, valid, reliable, and implemented consistently across all study participants?	yes	yes	yes	yes	yes	yes	yes	yes
12. Was loss to follow-up after baseline 20% or less?	yes	not applicable	no	not applicable	not reported	not applicable	not reported	not applicable
13. Were key potential confounding variables measured and adjusted statistically for their impact on the relationship between exposure(s) and outcome(s)?	yes	yes	yes	yes	yes	yes	yes	yes
Overall quality judgement	fair	good	good	good	good	fair	fair	good
